# The Antidiabetic Activities of Neocryptotanshinone: Screened by Molecular Docking and Related to the Modulation of PTP1B

**DOI:** 10.3390/nu14153031

**Published:** 2022-07-24

**Authors:** Jie Hao, Zhiming Qian, Zijian Liu, Guirong Zhang, Di Wang, Weiwei Han

**Affiliations:** 1 School of Life Sciences, Jilin University, Changchun 130012, China; haojie19@mails.jlu.edu.cn (J.H.); qianzm1321@mails.jlu.edu.cn (Z.Q.); zijianl20@mails.jlu.edu.cn (Z.L.); zgr@jlu.edu.cn (G.Z.); 2 Engineering Research Center of Chinese Ministry of Education for Edible and Medicinal Fungi, Jilin Agricultural University, Changchun 130118, China

**Keywords:** virtual screening, molecular docking, NCTS, Diabetes mellitus, PTP1B

## Abstract

The aim of this study was to provide a practical experimental basis for the development of Neocryptotanshinone (NCTS) as an effective hypoglycemic drug and a theoretical method for the rapid screening of natural compounds with hypoglycemic effects. Molecular docking was used to screen the most suitable ligand. Hematoxylin and eosin, immunohistochemical staining, enzyme-linked immunosorbent assay and Western Blotting approved the hypoglycemic effect of NCTS. According to the free energy of binding, among 180 active compounds from the Traditional Chinese Medicine Integrated Database, NCTS was finally chose for investigation its hypoglycemic effects. In db/db mice, NCTS significantly reduced body weight and plasma glucose, improved glucose tolerance and levels of fasting plasma glucose and glycated hemoglobin A1c, and decreased insulin resistance after six-week administration. NCTS restored the pathological state in the liver of db/db mice and significantly decreased protein tyrosine phosphatase 1B (PTP1B) expression in the liver and muscle of db/db mice, which is related to the regulatory effect of NCTS on insulin receptor substrate 1. In conclusion, we successfully explored the hypoglycemic effect of NCTS in db/db mice via regulating the expression of PTP1B.

## 1. Introduction

Diabetes mellitus (DM), which is characterized by metabolic disturbances, affected more than 220 million people worldwide in 2011, and this number will double by 2030 [1]. Type II diabetes mellitus (T2DM) is the most common type of DM, accounting for approximately 95% of all diabetes cases [2]. T2DM is mainly caused by genetic and environmental factors, and/or their interaction, which leads to the dysfunction of skeletal muscle, liver, and adipose tissues to insulin and pancreatic β cells; for example, tissues fail to respond normally to insulin, and pancreatic β cells fail to produce enough insulin to compensate [3,4,5]. In T2DM, insulin resistance (IR) leads to the development of hyperglycemia, which causes oxidative stress and the activation of inflammatory pathways, ultimately increasing the morbidity and mortality of T2DM patients [3]. Protein tyrosine phosphatase 1B (PTP1B), encoded by the protein tyrosine phosphatase non-receptor type 1 (*PTPN1*) gene, terminates insulin signaling by dephosphorylating the insulin receptor and insulin receptor substrate (IRS) [6,7,8]. PTP1B is closely related to IR. *PTPN1*-deficient mice are more responsive to insulin and leptin [9,10], have lower blood glucose levels, and have more normal insulin levels [6]. Aging leads to increased levels of PTP1B in various tissues [11] and dephosphorylation of IRS1 in the liver, which inhibits insulin signaling [12]. PTP1B-deficient mice are more sensitive to insulin. In the livers of diabetic mice that were intraperitoneally treated with PTP1B antisense oligonucleotides, PTP1B and blood glucose levels were decreased [13]. In the liver of obese mice, short-term strength exercise improved insulin sensitivity by reducing PTP1B levels [14].

Accordingly, to overcome the development of IR, anti-inflammatory agents, such as salicylates, have been found to improve insulin sensitivity by inhibiting nuclear factor kappa-B (NF-κB) [15]. The main drugs currently used for the treatment of IR are thiazolidinediones, which can cause weight gain, fluid retention, fractures, and even heart failure in patients [16]. Biguanides (e.g., metformin) improve IR by promoting adenosine monophosphate-activated protein kinase signaling [17]. However, the efficacy of biguanides often decreases over time [18], and biguanides can cause lactic acidosis in patients [17]. Until now, at least four PTP1B inhibitors have been developed as clinical trial candidates, including ertiprotafib, ISIS 113715, ISIS-PTP1B_Rx_, and trodusquemine.

Since they have various pharmacological activities, natural compounds have been attracting increasing attention as a new research trend [19]. Unfortunately, it is difficult to individually screen the efficacy of millions of natural compounds using traditional biological detection assays. We aimed to investigate new inhibitors [chemical composition from the Traditional Chinese Medicine Integrated Database (TCMID) TCMID 2.0 (http://www.megabionet.org/tcmid/ (accessed on 02 November 2017))] and study the binding mode of these inhibitors [neocryptotanshinone (NCTS)] to PTP1B [Protein Data Bank (PDB) ID 5QGF] [20] by molecular docking in order to mutually validate and complement the experimental findings. The interaction of PTP1B with the active part of NCTS was analyzed by molecular docking, thus systematically revealing the mechanism of action of NCTS and PTP1B. The results could provide new ideas for screening PTP1B inhibitors from the drug library. The results also provide a reference for the interaction between small molecules and proteins, and the design of functional products enriched with proteins.

*Salvia miltiorrhiza* Bunge, a traditional Chinese herbal medicine, has been used to treat cardiovascular diseases [21]. NCTS, obtained from the root of *Salvia miltiorrhiza* Bunge, is a tanshinone with the structure of a diterpenoid [22]. Until now, only one study has reported that NCTS can inhibit lipopolysaccharide-induced inflammation in RAW264.7 macrophages by the suppression of NF-κB and inducible nitric oxide synthase (iNOS) signaling pathways [21]. However, the other effects of NCTS remain unclear. 

In this study, 180 ligands were docked to PTP1B, and the top ligand was selected. In BKS.Cg-+Lepr^db^/+Lepr^db^/JNju (db/db) mice presenting a leptin receptor mutation [23], the hypoglycemic activity of NCTS was confirmed, which was further found to be related to the improvement of IR via reduction of the expression of PTP1B.

## 2. Materials and Methods

### 2.1. High-Throughput Virtual Screening and Molecular Docking

The active compounds were extracted from the TCMID, and the 3D structures of 180 different ligands were downloaded from the PubChem database, including *Salvia miltiorrhiza*, Tennantine, Phellodendron, Chicory, Eleuthero, and Lycium, to form a virtual screening database based on relevant literature searched from the Chinese National Knowledge Infrastructure (CNKI) and Wanfang databases, as well as the English PubMed database. The crystal structure of PTP1B was downloaded from PDB (PDB ID: 5QGF) and the A chain was selected for bulk molecular docking using AutoDock vina [24]. The possible conformations of the protein receptors and inhibitors, as well as the binding free energy, were calculated according to the Lamarck GA genetic algorithm. Six compounds with the best docking effect on the PTP1B protein were selected for further analysis of interactions, and the most effective compound, NCTS, was identified by docking analysis with different sites of PTP1B.

### 2.2. Animal Feeding and NCTS Administration

NCTS (CAS NO.: 109664-02-0, purity: HPLC ≥ 98%) purchased from Chengdu Desite Biotechnology Co., Ltd., Chengdu, China, and Metformin Hydrochloride (Met) (CAS NO.: 1115-70-4, National Drug Code: H20023370) purchased from Sino-American Shanghai Squibb Pharmaceutical Co., Ltd., Shanghai, China, were dissolved in 0.9% normal saline containing 0.1% dimethyl sulfoxide (DMSO) (DH105-2, Beijing Dingguo Changsheng Biotechnology Co., Ltd., Beijing, China) and 0.6% Tween 80 (CAS NO.: 9005-65-6, T104866, Shanghai Aladdin Bio-Chem Technology Co., Ltd., Shanghai, China).

Animal experiments were approved by the Animal Ethics Committee of Jilin University (SY202105006). The db/db mice (male, 10–11 weeks old) and their littermates with genotype BKS.Cg-Dock7^m^+/Dock7^m^+/JNju (m/m) used as normal controls (male, 10–11 weeks old) were purchased from Changzhou Cavens Laboratory Animal Co., Ltd. [Changzhou, Jiangsu, China, SCXK (SU) 2018-0002] and were housed in a controlled room with a stable and continuous temperature (23 ± 1 °C) and humidity (40–70%) under a 12-h light/dark cycle without convective wind. Mice were fed a normal chow diet (maintenance feed of SPF grade; Liaoning Changsheng Biotechnology Co., Ltd., Liaoning, China) for six weeks.

After one week of adaptive feeding, 18 db/db mice were randomly divided into three groups (*n* = 6 per group) and orally treated with 10 mg/kg of NCTS (NCTS-treated db/db mice), 100 mg/kg of Met (Met-treated db/db mice), and 3.33 mL/kg of normal saline (containing 0.1% DMSO and 0.6% Tween-80) (vehicle-treated db/db mice) once a day for another six weeks. Another six m/m mice were orally treated with 3.33 mL/kg of normal saline (containing 0.1% DMSO and 0.6% Tween-80) (serving as vehicle-treated m/m mice) once a day for six weeks. Body weights and plasma glucose levels were monitored weekly. At the end of the experiment, all mice were euthanized by anesthesia with carbon dioxide. Serum samples were collected from the tail vein, and organs including the heart, liver, spleen, lung, kidney, skeletal muscle, and pancreas were collected for visceral index calculations and biochemical and pathological analyses. The percentage of the organ weight to the body weight was defined as the organ indices.

### 2.3. Oral Glucose Tolerance Test (OGTT)

After the latter agent’s administration, all mice were fasted for 12 h and then an OGTT was carried out. A glucose solution (2.0 g/kg) (B21882, Shanghai Yuanye Bio-Technology Co., Ltd., Shanghai, China) was rapidly orally administered to all mice after measuring the fasting plasma glucose values. Blood samples were collected from the tail vein of mice at 0, 30, 60, 120, and 240 min after glucose administration. The plasma glucose content was measured using a detection kit (ADS-W-TDX001, Jiangsu Feiya Biotechnology Co., Ltd., Jiangsu, China) using the glucose oxidase peroxidase (GOPOD) oxidase method.

The area under the blood glucose curve (AUC) was used to evaluate the glucose tolerance. AUC was determined using the following formula: AUC = (glycemia 0 h + glycemia 0.5 h) × 0.25 + (glycemia 0.5 h + glycemia 1 h) × 0.25 + (glycemia 1 h + glycemia 2 h) × 0.5.

### 2.4. Food and Water Intake Measurement

Food and water intake can reflect the metabolic status of mice. The food and water intake of all mice at 24 h was measured using metabolic cages after the last drug administration.

### 2.5. Hematoxylin and Eosin (H&E) Staining

The liver was fixed in 4% paraformaldehyde (BL539A, Biosharp, Guangzhou, China), and the muscle was fixed in muscle tissue fixative (G1111, Servicebio, Wuhan, China). The fixed tissues were dehydrated via continuous immersion in 30%, 50%, 70%, 80%, 95%, and 100% ethanol. The dehydrated tissues were then immersed in xylene, embedded in wax, and cut into 5 μm sections. Paraffin sections were deparaffinized in xylene, anhydrous ethanol, and 75% alcohol following the initial preparation and stained with H&E. The histopathological state of the liver and muscle was observed after another dehydration process using an upright optical microscope (Nikon Eclipse E100; Nikon, Japan).

### 2.6. Enzyme-Linked Immunosorbent Assay (ELISA) Analyses

The serum levels of insulin and glycated hemoglobin A1c (GHbA1c) were measured using commercial ELISA kits (RX202485M and RX202603M) (Quanzhou Ruixin Biotechnology Co., Ltd., Fujian, China).

Homeostasis model assessment (HOMA) is a mathematical model that can be used to assess IR [25]. The homeostasis model of insulin resistance (HOMA-IR) was calculated according to the following formula: HOMA-IR = [(fasting insulin (μIU/mL)) × (fasting glucose (mmol/L))]/22.5.

### 2.7. Western Blotting

Proteins were extracted from the liver and muscle using radioimmunoprecipitation assay (RIPA) lysis buffer (10×, 20-188, Merck Millipore, Billerica, MA, USA) containing a protease and phosphatase inhibitor cocktail (P002, New Cell & Molecular Biotech Co., Ltd., Suzhou, China). The protein concentrations were determined using the Pierce^TM^ BCA Protein Assay Kit (23225, Thermo Scientific^TM^, Waltham, MA, USA).

Equal amounts of protein samples were separated by 12% sodium dodecyl sulfate polyacrylamide gel electrophoresis according to molecular size. The isolated proteins were completely transferred to polyvinylidene difluoride (PVDF) membranes (0.45 μm) (10600023, Cytiva, Marlborough, MA, USA). The loaded membranes were incubated with fast sealing fluid (GF1815, genefist, Oxfordshire, UK) at 4 °C for 30 min, and then incubated at 4 °C overnight with the following primary antibodies: phospho (P)-insulin receptor substrate 1 (IRS1) (dilution: 1:2000) (AP0552) (ABclonal Technology Co., Ltd., Wuhan, China), total (T)-IRS1 (dilution: 1:2000) (A16902), P-Protein Kinase B (AKT) (dilution: 1:2000) (AP1068), T-AKT (dilution: 1:1000) (A18120), PTP1B (dilution: 1:2000) (A1590), α-actin (dilution: 1:2000) (AF5332) (Jiangsu Kinke Biological Research Center Co., Ltd., Jiangsu, China), and β-actin (dilution: 1:2000) (sc-47778) (Santa Cruz Biotechnology (Shanghai) Co., Ltd., Shanghai, China). The membranes were incubated with peroxidase/horseradish peroxidase (HRP)-conjugated goat anti-rabbit IgG (H+L) (E-AB-1003) or goat anti-mouse IgG (H+L) (E-AB-1001) (Elabscience Biotechnology Co., Ltd., Wuhan, China) at 4 °C for 4 h. After washing, the membranes were immersed in NcmECL Ultra (P10200; New Cell and Molecular Biotech Co., Ltd., Suzhou, China). Immunoblots were visualized using an imaging system (Tanon 5200; Shanghai Tianneng Technology Co., Ltd., Shanghai, China) and quantified using ImageJ software (National Institutes of Health, Bethesda, MD, USA).

### 2.8. Immunohistochemical Examination

Consistent with the H&E staining protocol, we recovered the antigen and blocked the endogenous peroxidase activity in the paraffin sections of the liver and muscle. Then, 3% bovine serum albumin (BSA) was added to evenly cover the tissues, and they were sealed for 30 min at room temperature. The tissues were incubated with PTP1B (A1590; ABclonal Technology Co., Ltd., Wuhan, China) at 4 °C overnight. Subsequently, the tissues were incubated with peroxidase/HRP-conjugated goat anti-rabbit IgG (H+L) (E-AB-1003; Elabscience Biotechnology Co., Ltd., Wuhan, China) at room temperature for 50 min. Paraffin sections were reacted with diaminobenzidine (DAB) chromogenic reagent (DA1016; Solarbio, Beijing, China). After terminating the reaction, hematoxylin was used to counterstain the nuclei. The sections were dehydrated and sealed for observation under a microscope and a quantitative analysis was performed to determine the positive state of PTP1B in the tissues.

### 2.9. Statistical Analysis

All values are expressed as the mean ± Standard Deviation (S.D.). One-way analysis of variance (ANOVA) followed by the post-hoc multiple comparisons (Tukey’s) test was used to determine differences using SPSS Statistics 26 (BONC, Beijing, China). Statistical significance was defined as a *p* value less than 0.05. AutoDock Vina software was used for docking. Protein–ligand interactions were shown by PyMOL.

## 3. Results

### 3.1. High-Throughput Virtual Screening and Molecular Docking

**Figure 1:**
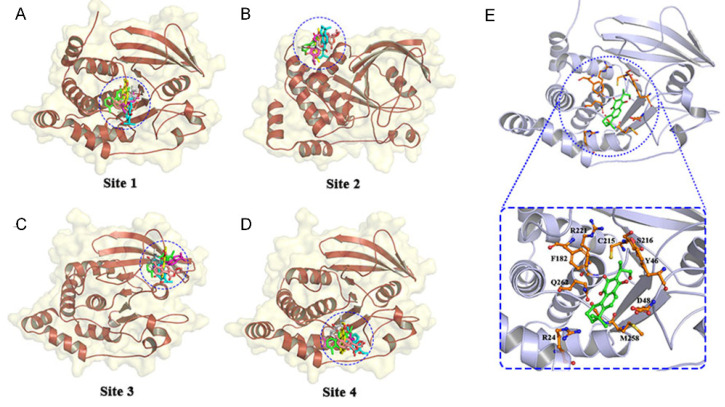
Molecular docking of the top six ligands and sites of the protein tyrosine phosphatase 1B (PTP1B). Six compounds located at (**A**) site 1, (**B**) site 2, (**C**) site 3, and (**D**) site 4. (**E**) The active residues around ligand binding in site 1.

The affinity scores of 180 ligands for PTP1B were ranked, and the top 50 ligands were selected. The lower of the free energy of binding obtained by docking is, the more suitable the binding of the inhibitor to PTP1B is. PTP1B contains both active and allosteric sites. To obtain suitable binding sites for the complexes, we tried to dock the top six ligands in terms of docking score to each site of the receptor protein (Figure 1A–D). The binding free energy values were shown in Table 1, and the docking positions were shown in Figure 1. The results showed that the lowest binding free energy values were obtained when the six ligands were docked to the site-1 site, and among the six compounds, the NCTS had the best effect. Therefore, NCTS can be regarded as a competitive inhibitor located at the active site, and its detailed interaction with the active site is shown in Figure 1E.

### 3.2. Effect of NCTS on Hypoglycemia in db/db Mice

**Figure 2:**
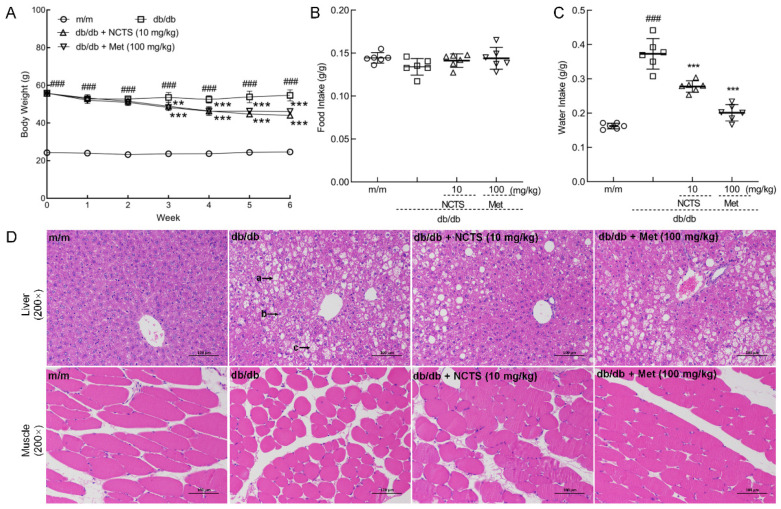
The effect of Neocryptotanshinone (NCTS) on hypoglycemic in db/db mice. (**A**) NCTS suppressed bodyweight of db/db mice. (**B**) NCTS had no effect on food intake, but (**C**) reduced the water intake of db/db mice. (**D**) Histopathological observation of liver and muscle tissues of db/db mice (200×, scale bar: 100 μm) (a. steatosis of hepatocytes and circular vacuoles of varying sizes in the cytoplasm, b. the nucleus of the hepatocyte is centered, c. hepatocytes showing ballooning degeneration.) Data are presented as the mean ± Standard Deviation (S.D.) (*n* = 6) and analyzed via a one-way analysis of variance (ANOVA) test followed by post-hoc Tukey’s multiple comparison tests. ^###^ *p* < 0.001 vs. vehicle-treated m/m mice; ** *p* < 0.01 and *** *p* < 0.001 vs. vehicle-treated db/db mice.

Compared with vehicle-treated db/db mice, both NCTS and Met strongly suppressed body weight (*p* < 0.01, Figure 2A) without influencing food intake (Figure 2B). Both NCTS and Met reduced the water intake of db/db mice (*p* < 0.001, Figure 2C), suggesting an improvement in diabetic symptoms. NCTS enhanced the organ indices, including those for the pancreas, heart, kidney, and lung (*p* < 0.001, Table 2), decreased the organ indices of liver (*p* < 0.01, Table 2), but not for the spleen; meanwhile, Met enhanced the organ indices, including those of the pancreas (*p* < 0.001), heart (*p* < 0.001), and lung (*p* < 0.01) in db/db mice, and decreased the organ indices of liver (*p* < 0.01) (Table 2).

The protective effect of NCTS in the liver was confirmed by H&E staining. In the livers of db/db mice, a large number of hepatocytes with balloon-like degeneration, centered nuclei, vacuolated cytoplasm, a small number of hepatocytes with steatosis, round vacuoles of different sizes in the cytoplasm, and a small number of hepatocytes with intranuclear inclusion bodies were noted. All of these pathological changes improved after NCTS and Met treatment (Figure 2D), and the relative size of lipid droplets were significantly reduced with the action of NCTS or Met (*p* < 0.05, Figure S1A). Compared with vehicle-treated db/db mice, the low muscle fiber cross-sectional areas of skeletal muscle were enhanced in both NCTS and Met-treated mice (*p* < 0.001, Figure 2D and Figure S1B).

**Figure 3:**
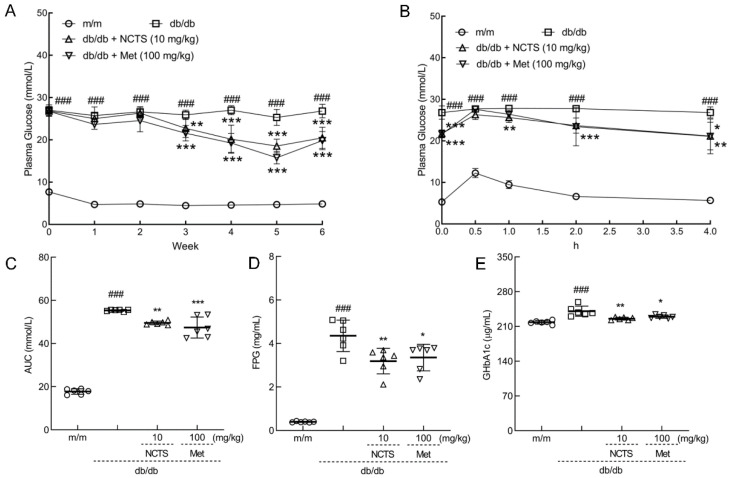
The effect of NCTS on hypoglycemia in db/db mice. (**A**) NCTS suppressed the enhancement of plasma glucose. (**B**) NCTS improved glucose tolerance demonstrated by Oral Glucose Tolerance Test (OGTT) experiment and was quantified by (**C**) the area under the blood glucose curve (AUC). NCTS suppressed the serum levels of (**D**) fasting plasma glucose (FPG) and (**E**) glycated hemoglobin A1c (GHbA1c). Data are presented as the mean ± S.D. (*n* = 6) and analyzed via a one-way ANOVA test followed by post-hoc Tukey’s multiple comparison tests. ^###^ *p* < 0.001 vs. vehicle-treated m/m mice; * *p* < 0.05, ** *p* < 0.01 and *** *p* < 0.001 vs. vehicle-treated db/db mice.

Compared to vehicle-treated db/db mice, both NCTS and Met suppressed the enhancement of plasma glucose after the three-week treatment and continued until the end of the experiment (*p* < 0.01, Figure 3A). Following a 4-h administration of 2 g/kg of glucose, the plasma glucose levels of db/db mice were stable and significantly higher than those of m/m mice (*p* < 0.001, Figure 3B). Both NCTS and Met decreased plasma glucose levels from 30 min after glucose administration to 4 h (*p* < 0.05, Figure 3B). The AUC was significantly reduced in both Met- and NCTS-treated mice compared with that in db/db mice (*p* < 0.01, Figure 3C). Compared with vehicle-treated db/db mice, NCTS and Met significantly suppressed the serum levels of fasting plasma glucose (FPG) (*p* < 0.05, Figure 3D) and GHbA1c (*p* < 0.05, Figure 3E), confirming the effect of NCTS on hyperglycemia. 

### 3.3. NCTS-Modulated IR Related to PTP1B in db/db Mice

**Figure 4:**
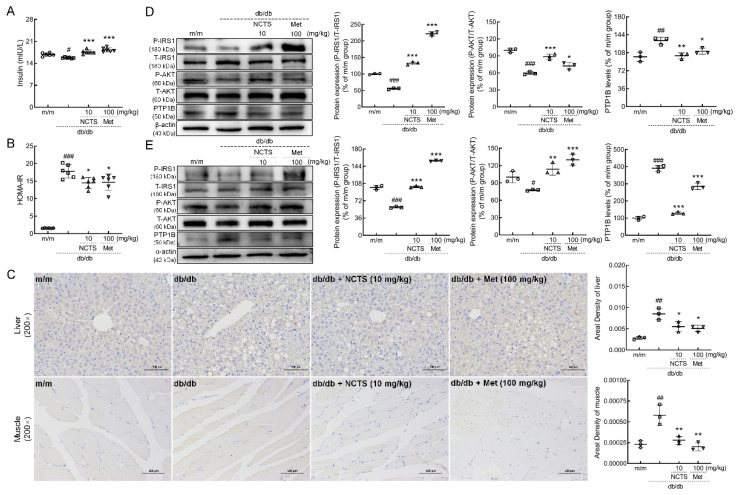
The effect of NCTS on modulation of insulin resistance (IR) related to PTP1B in db/db mice. (**A**) NCTS enhanced the serum levels of insulin (*n* = 6). (**B**) NCTS decreased the value of homeostasis model of insulin resistance (HOMA-IR) (*n* = 6). (**C**) NCTS suppressed the expressions of PTP1B demonstrated by immunohistochemistry (*n* = 3). NCTS enhanced the phosphorylation of insulin receptor substrate 1 (IRS1) and protein kinase B (AKT) and suppressed the expressions of PTP1B in the (**D**) liver and (**E**) muscle of db/db mice (*n* = 3). Quantification data were normalized to α/β-actin or the corresponding total protein concentration, and expressed as the percentage of m/m mice. Data are presented as the mean ± S.D. and analyzed via a one-way ANOVA test followed by post-hoc Tukey’s multiple comparison tests. ^#^ *p* < 0.05, ^##^ *p* < 0.01 and ^###^ *p* < 0.001 vs. vehicle-treated m/m mice; * *p* < 0.05, ** *p* < 0.01 and *** *p* < 0.001 vs. vehicle-treated db/db mice.

NCTS and Met administration strongly enhanced the serum levels of insulin (*p* < 0.001, Figure 4A) and significantly decreased the value of HOMA-IR (*p* < 0.05, Figure 4B) in db/db mice, thereby indicating the anti-IR effects of NCTS.

In both the liver and muscle of db/db mice, NCTS and Met strongly suppressed the expression of PTP1B (*p* < 0.05, Figure 4C). Compared with vehicle-treated db/db mice, NCTS enhanced the expression of P-IRS1 (*p* < 0.001) and P-AKT (*p* < 0.01) and reduced the expression levels of PTP1B (*p* < 0.01) in the liver (Figure 4D) and muscle (Figure 4E). Similar results were observed in the Met-treated db/db mice.

## 4. Discussion

Natural compounds are regarded as a source of great potential in the field of drug development. The natural compounds improving IR derived from plants mainly include three categories: flavonoids, terpenes, and phenylpropanoids [26]. In this study, 180 ligands from the TCMID database were docked onto PTP1B. The top six ligands docked to both the active and allosteric sites. Among the six compounds, NCTS had the best effect; therefore, it can be regarded as a competitive inhibitor that is located at the active site. In db/db mice, the hypoglycemic activity of NCTS was analyzed by changes in body weights, food and water intake, OGTT, and the levels of GHbA1c. Weight loss may reduce the degree of IR, which may be mediated by reducing the size of fat cells, and the same results were noted in NCTS-treated db/db mice. Muscle loss is associated with the degradation of muscle proteins that occurs in diabetes [27], which is accompanied by the occurrence of IR. NCTS and Met significantly increased the cross-sectional area of muscle fibers in the skeletal muscle of db/db mice. Therefore, the anti-IR effects of NCTS were analyzed based on the levels of insulin and the expression of PTP1B and its related proteins.

A typical symptom of diabetes in mice is a change in drinking and urinating patterns. The six-week administration of NCTS significantly reduced the water intake of db/db mice, suggesting an improvement in hyperglycemia symptoms. The OGTT is an important criterion for diagnosing diabetes. NCTS significantly suppressed plasma glucose levels in the OGTT, suggesting an association between NCTS and glucose tolerance. Glucose tolerance can be enhanced by reducing IR [28], and the knockout of PTP1B helps to improve high-fat, diet-induced poor glucose tolerance [29]. FPG can be used to evaluate the plasma glucose regulation process, and elevated FPG levels indicate the failure of glycemic regulation [30]. GHbA1c is the product of a non-enzymatic reaction of plasma glucose and hemoglobin in the body [31]. Since this process is irreversible, GHbA1c can objectively reflect the plasma glucose concentration in mice. NCTS showed hypoglycemic activity related to its anti-IR. Insulin, which is secreted by pancreatic β-cells, plays a crucial role in the occurrence and development of diabetes. Insufficient insulin secreted by pancreatic β cells cannot compensate for IR, which easily leads to the occurrence of T2DM [32]. IR can be directly assessed by HOMA-IR values [33,34], and a high HOMA-IR value can reflect a lower insulin sensitivity or a high level of IR [35]. NCTS remarkably enhanced the serum levels of insulin, consequently reducing the value of HOMA-IR in db/db mice and confirming its anti-IR activity. 

PTP1B is a specific non-receptor protein tyrosine phosphatase (PTPase) with a unique structure that can promote the dephosphorylation of related proteins such as insulin receptor and IRS during insulin action, thus acting as a negative regulator of insulin receptor signaling [36]. PTP1B inhibitors can effectively reduce the value of HOMA-IR, ultimately improving glucose intolerance and hyperglycemia [37]. NCTS reduced the expression levels of PTP1B in the liver and muscle of db/db mice. 

Insulin plays a central role in the maintenance of glucose homeostasis. Insulin receptors are phosphorylated and activated after binding to insulin. IRS1 acts upstream of AKT [38], and the phosphorylation of IRS1 subsequently activates phosphatidylinositol 3-kinase (PI3K)/AKT [39], thereby activating the insulin signaling pathway, enhancing glucose uptake, lowering plasma glucose levels, and increasing glycogen and protein synthesis [40]. The phosphorylation level of IRS1 can be increased by inhibiting the expression of PTP1B [41]. In the presence of inhibition of PTP1B expression, the phosphorylation of IRS1 increases, leading to the interaction of IRS1 with the p85 regulatory subunit of PI3K and resulting in the subsequent activation of AKT [40]. 

At present, the research on NCTS is still in the preliminary stage. NCTS can achieve anti-inflammatory effects by inhibiting NF-κB and iNOS signaling pathways in lipopolysaccharide-induced RAW264.7. Our study proves the hypoglycemic efficacy of NCTS. Comparatively, chronic and high-dose administration with Met may show side effects on the gastrointestinal tract [42], and its high hydrophilicity leads to a reduced degree of metabolic absorption and insufficient bioavailability [43,44]. However, the security of NCTS needs further investigation. On the other hand, IR is reported to be related to the dysfunction of the transport of glucose transporter 4 (GLUT4) [26]. However, based on our present study, the effects of NCTS on GLUT4 failed to be detected, which still needs further investigation. Furthermore, the roles of PTP1B and IRS1 in NCTS-mediated hypoglycemic effects should be further confirmed in in vitro and in vivo experiments.

## 5. Conclusions

In conclusion, we successfully explored the hypoglycemic effect of NCTS in db/db mice related to the expression of PTP1B. This study provides a theoretical method for the rapid screening of natural compounds with hypoglycemic effects. This research is bound to play a beneficial role in the establishment of natural product libraries with improved insulin resistance and the screening of clinical drugs for human T2DM.

## Figures and Tables

**Table 1:** The docking score of six compounds.

Compound	Free Energy (kcal/mol)			
	Site 1	Site 2	Site 3	Site 4
1,2-Dihydrotanshiiquinone	−6.1	−5.2	−6.0	−5.4
NCTS	−6.2	−5.9	−5.3	−5.1
Dehydromiltirone	−5.4	−5.3	−5.2	−5.2
Tanshinonel	−6.1	−5.9	−5.3	−5.6
Danshen Spiroketallactone	−6.0	−5.5	−5.2	−5.1
Neotanshinone	−5.6	−5.3	−5.3	−5.2

NCTS: Neocryptotanshinone.

**Table 2:** The effects of NCTS on organ indices of db/db mice.

Organs (%)	m/m	db/db	db/db + NCTS (10 mg/kg)	db/db + Met (100 mg/kg)
Liver	4.046 ± 0.279	6.866 ± 0.184 ^###^	5.925 ± 0.687 **	5.874 ± 0.282 **
Pancreas	1.218 ± 0.073	0.319 ± 0.047 ^###^	0.525 ± 0.037 ***	0.585 ± 0.072 ***
Heart	0.517 ± 0.025	0.254 ± 0.022 ^###^	0.355 ± 0.023 ***	0.332 ± 0.031 ***
Spleen	0.244 ± 0.019	0.136 ± 0.012 ^###^	0.147 ± 0.028	0.132 ± 0.015
Kidney	1.255 ± 0.016	0.793 ± 0.067 ^###^	1.020 ± 0.076 ***	0.864 ± 0.075
Lung	0.537 ± 0.025	0.245 ± 0.021 ^###^	0.330 ± 0.017 ***	0.307 ± 0.043 **

The data were analyzed using a one-way ANOVA and expressed as means ± S.D. (*n* = 6). ^###^ *p* < 0.001 vs. vehicle-treated m/m mice; ** *p* < 0.01 and *** *p* < 0.001 vs. vehicle-treated db/db mice.

## Data Availability

Not applicable.

## Supplementary Materials

The following supporting information can be downloaded at: https://www.mdpi.com/article/10.3390/nu14153031/s1, Figure S1: (**A**) The relative size of lipid droplets and (**B**) quantification of the muscle fiber cross-sectional areas, according to representative H&E staining results in Figure 2D (*n* = 3). Data are presented as the mean ± S.D. and analyzed via a one-way ANOVA test followed by post-hoc Tukey’s multiple comparison tests. ^###^ *p* < 0.001 vs. vehicle-treated m/m mice; * *p* < 0.05, ** *p* < 0.01 and *** *p* < 0.001 vs. vehicle-treated db/db mice.
