# Supplementary material for: The Antidiabetic Activities of Neocryptotanshinone: Screened by Molecular Docking and Related to the Modulation of PTP1B
Source: Nutrients. 2022 Jul 24;14(15):3031. doi: 10.3390/nu14153031 (PMC9330310; doi:10.3390/nu14153031)
Supplement: Supplementary file 1 [file nutrients-14-03031-s001.zip › nutrients-1828172-supplementary.pdf]

Supplementary Materials

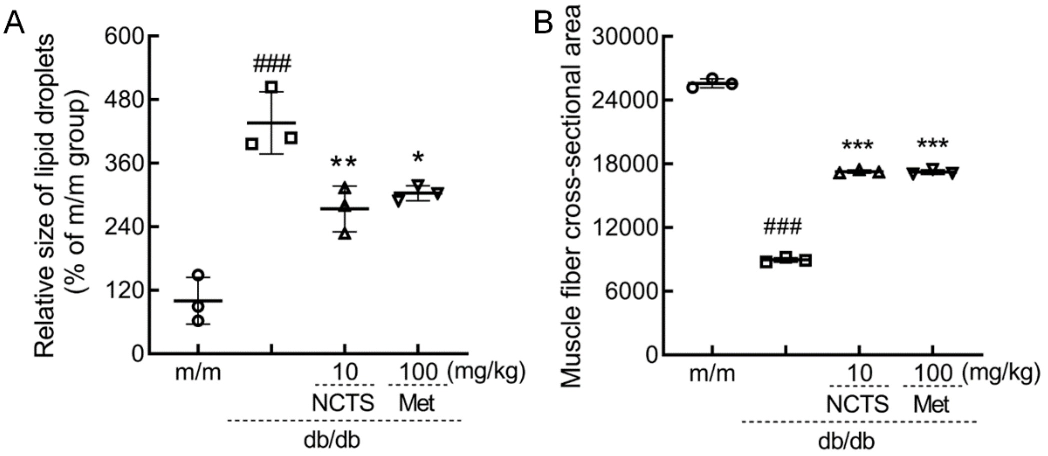

Figure S1. (A) The relative size of lipid droplets and (B) quantification of the muscle fiber cross-sectional areas, according to representative hematoxylin and eosin (H&E) staining results in Figure 2D ($n = 3$). Data are presented as the mean ± S.D. and analyzed via a one-way ANOVA test followed by post-hoc Tukey's multiple comparison tests. $^{###}p < 0.001$ vs. vehicle-treated m/m mice; $^{*}p < 0.05$, $^{**}p < 0.01$ and $^{***}p < 0.001$ vs. vehicle-treated db/db mice.
